# Supplementary material for: Identification of photoperiod-induced specific miRNAs in the adrenal glands of Sunite sheep (Ovis aries)
Source: Front Vet Sci. 2022 Jul 22;9:888207. doi: 10.3389/fvets.2022.888207 (PMC9354845; doi:10.3389/fvets.2022.888207)
Supplement: Supplementary file 1 [file Data_Sheet_1.ZIP › Supplementary file/miRDeep2 file.zip!/miRDeep2 file/Novel_Structure/SPLP42A1/Novel_128.pdf]

Provisional ID	:	NC_019478.2_26829
Score total	:	1.3
Score for star read(s)	:	-1.3
Score for read counts	:	-0.3
Score for mfe	:	1.4
Score for randfold	:	1.6
Score for cons. seed	:	
Total read count	:	11
Mature read count	:	11
Loop read count	:	0
Star read count	:	0

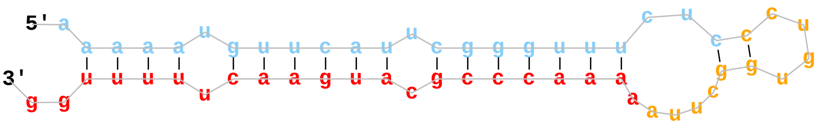

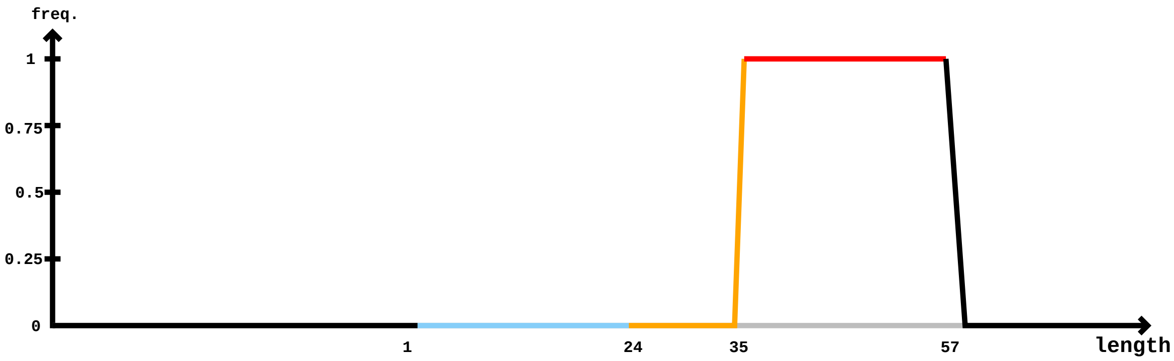


```
                                                       Star                                    Mature
5'- gucacagaaaucaaccaacuuguuaucggguuagccaaaaauguucauucggguuucucccuguggcuuaaaaacccgcaugaacuuuuuggccaacccaacacauuccgca   -3'    exp
    ....................((((...(((((.((((((((.((((((.(((((((..((....)).....))))))).))))))))))))))).)))))))))).........    reads      mm         sample
    ......................................................................aaaacccgcaugaacuuuuugg....................    11         0          seq
```
